# Single-Stage Image-Guided Percutaneous Ablation with Thoracoscopic Resection for Multiple Pulmonary Lesions in a Hybrid Operating Room: A Retrospective Study

**DOI:** 10.3390/cancers16203512

**Published:** 2024-10-17

**Authors:** Ling-Kai Chang, Po-Keng Su, Pak-Si Chan, Shwetambara Malwade, Wen-Yuan Chung, Shun-Mao Yang

**Affiliations:** 1 Interventional Pulmonology Center, National Taiwan University Hospital, Hsin-Chu Branch, Hsinchu 300195, Taiwan; nathanchang100@gmail.com (L.-K.C.); spk911212@gmail.com (P.-K.S.); percychan1214@gmail.com (P.-S.C.); lex780117@gmail.com (W.-Y.C.); 2 Department of Internal Medicine, National Taiwan University Hospital, Hsin-Chu Branch, Hsinchu 300195, Taiwan; 3 Department of Surgery, National Taiwan University Hospital, Hsin-Chu Branch, Hsinchu 300195, Taiwan; shwetambara.malwade@siemens-healthineers.com; 4 Department of Anesthesiology, National Taiwan University Hospital, Hsin-Chu Branch, Hsinchu 300195, Taiwan; 5 Department of Advanced Therapies, Siemens Healthcare Limited, Taipei City 11503, Taiwan; 6 Department of Traumatology, National Taiwan University Hospital, Hsin-Chu Branch, Hsinchu 300195, Taiwan

**Keywords:** lung cancer, hybrid operating room, surgical resection, ablation, multiple lung lesions

## Abstract

Background: Different approaches are required in treating patients with multiple pulmonary lesions. A multistage procedure may increase the risk of complications and patient discomfort. This study reports an initial experience with single-stage management of multiple lung lesions using percutaneous ablation with thoracoscopic resection in a hybrid operating room (HOR). Methods: We retrospectively evaluated patients who underwent combined ablation and resection in an HOR between May 2022 and July 2024. All patients received a single anesthesia via endotracheal tube intubation. The clinical data, operative findings, and pathological characteristics of the lung nodules were recorded. Results: A total of 22 patients were enrolled in this study. Twenty patients underwent unilateral procedures, while the other two patients underwent bilateral procedures. Ablations were performed before lung resection in 21 patients; only 1 patient underwent surgery first. The median global operating room time was 227.0 min. The median total radiation dose (dose area product) was 14,076 μGym^2^. The median hospital postoperative length of stay was 2 days. Conclusions: The single-stage procedure of percutaneous ablation with thoracoscopic resection under general anesthesia in an HOR is feasible and safe. This procedure is an alternative method for managing multiple pulmonary lesions.

## Simple Summary

Following technological advancements, there has been a rise in the early detection of pulmonary nodules, with more patients found to have multiple lesions. These lesions may indicate benign or malignant stages, each requiring careful evaluation and management. Multiple treatment strategies can be followed while ensuring patient comfort and minimizing complications. Thus, our study aims to provide an example of single-stage management for multiple lung lesions, using percutaneous ablation and thoracoscopic resection in a hybrid operating room (HOR). These combined procedures in a single stage and setting allows for a minimally invasive experience for patients, with minimal complications and a shorter operation time. In addition to personalized therapy, there is more flexibility for managing complications in the HOR. Thus, these initial results indicate a feasible and safe single-stage workflow and an alternative way to manage multiple pulmonary lesions in patients.

## 1. Introduction

Lung cancer is a leading cause of death worldwide. Recently, pulmonary nodules have been frequently detected during low-dose computed tomography (CT) screenings [1]. With increased early detection, the early management of lung lesions allows for better survival of certain patient groups [2,3]. This demands a more efficient curative treatment, especially for suspected malignant cases [4]. Despite the numerous methods available for the management of pulmonary nodules [5,6,7,8,9], surgical resection is the most often used method [10,11]; however, only approximately 30% of patients are potential surgical candidates because of cardiopulmonary limitations, advancing age, and the presence of other comorbidities [12,13].

Recently, an increasing number of patients have been diagnosed with multiple lesions [14]. Multiple scattered pulmonary nodules may indicate early-stage multiple lung cancers or several stages of lung cancer, from benign to atypical to malignant [5,14]. Regardless of the presence of isolated or multiple lung lesions, the evaluation and management of benign and malignant lesions are common concerns that need to be addressed, as they significantly impact clinical treatment strategies and could lead to unnecessary surgeries for noncancerous lesions [14]. For instance, surgical excision may involve sacrificing a large lung volume when the lesion is located in the central zone. These aspects have led to the development of alternative nonsurgical interventions, such as stereotactic body radiation therapy (SBRT) or proton therapy. However, these therapies involve high radiation doses, with potential damage to the surrounding tissue, chest wall pain, or skin damage [15]. Further advancements have led to the emergence of percutaneous ablative therapies, such as radiofrequency ablation (RFA), microwave ablation (MWA), cryoablation, and laser ablation [16,17,18,19,20]. Clinically, thermal ablation has several advantages, including procedural safety and preservation of lung function [16,17,18,21,22,23].

There are several existing clinical guidelines for thermal ablation, including those published by the American College of Chest Physicians [24], the National Comprehensive Cancer Network [5], and the Cardiovascular and Interventional Radiology Society of Europe [25,26]. It is essential to adhere to these guidelines for the optimal management of lung lesions. MWA is a heat-based ablation technique involving a lower heat-sink effect and related pain than RFA [27]. Various factors must be considered when using MWA, including the method of monitoring the ablation zone during the procedure, as well as the choice of MWA needle brands for different shapes and ablation zones [28,29,30,31,32]. Similarly, cryoablation is another emerging minimally invasive technique for malignant lung nodules and an alternative surgical treatment method [33]. A cross-sectional assessment of the post-ablation zones following cryoablation is easy to perform. Moreover, it is an option for lung nodules with ground glass opacities [34]. MWA is a more time-efficient method that helps preserve the lung parenchyma, shape, and function; however, it may result in significant pain when managing lesions adjacent to the pleura. As mentioned in one guideline, thermal ablation does not preclude subsequent treatment options, such as surgical resection [35,36]. In these cases, wedge resection is easier to perform than ablation. Management of such multiple existing lesions can be performed simultaneously and independently.

The development of a hybrid operating room (HOR) is an important advancement, especially in supporting the management of pulmonary nodules [37]. Real-time and high-definition imaging guidance during thoracic surgical procedures has improved existing techniques [38]. Two-dimensional fluoroscopy and three-dimensional cone-beam computed tomography (CBCT) in the HOR can aid appropriate device navigation and positioning [39]. Furthermore, invasive thoracic surgical procedures involving ablation and resection can be efficiently performed under general anesthesia (GA) with single-lung ventilation. The HOR provides the necessary infrastructure for combined procedures in the same suite and is suitable for the appropriate treatment of different lesions, along with a tailored approach [40]. Here, we present our initial experience of using the HOR for the management of multiple lung lesions with a single-stage procedure combining percutaneous ablation and thoracoscopic resection.

## 2. Materials and Methods

### 2.1. Study Design and Patients

We retrospectively evaluated consecutive patients who underwent combined image-guided percutaneous lung ablation and thoracoscopic lung resection in an HOR at the National Taiwan University Hospital, Hsinchu Branch, between May 2022 and July 2024. This study was approved by the Institutional Review Board of the National Taiwan University Hospital Hsinchu Branch (approval number: 202408097RINA). The indications for curative treatment of multiple lung lesions were as follows: (1) pathologically confirmed primary or metastatic lung cancers or (2) persistence of a subsolid nodule on follow-up CT with highly suspicious malignancy or precancerous lesions. Centrally located lesions were considered for ablation, and peripheral lesions were considered for resection. The decision was made by a multidisciplinary team comprising a thoracic oncologist, a chest surgeon, and an interventional pulmonologist.

### 2.2. Anesthesia and Surgical Preparation

The entire single-stage procedure of combined image-guided ablation and thoracoscopic resection was performed in an HOR equipped with a robotic C-arm CBCT system (ARTIS pheno; Siemens Healthcare GmbH). All patients underwent GA via double-lumen endotracheal tube intubation or a single-lumen tube with an endobronchial blocker. The fraction of inspired oxygen (FiO_2_) was maintained under 40% to prevent lung collapse. According to the location of the lung lesion, the patients were positioned in the supine, prone, or lateral decubitus position according to the optimal access route for insertion of the ablation needle. In this study cohort, we used the Emprint™ ablation system (Medtronic, Minneapolis, MN, USA) or Hi-Sphere 16 G/20 cm (ECO Medical Instruments Co., Ltd., Nanjing, China) for microwave ablation and the CryoCare System (Endocare, Inc., Irvine, CA, USA) for cryoablation.

### 2.3. Image-Guided Lung Ablation

**Figure 1:**
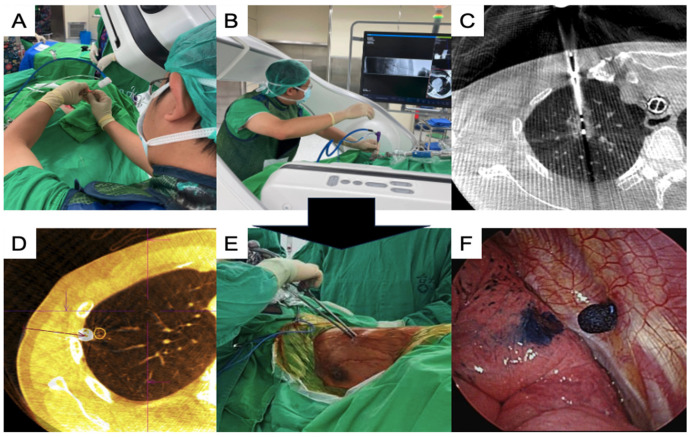
Single-stage synchronized procedures of ablation and VATS in an HOR. (**A**) The C-arm projects the laser cross to insert the coaxial needle for the ablation procedure; (**B**) insertion of the ablation needle using arm support under a progressive view augmented fluoroscopy; (**C**) post-ablation CT showing the ablation zone while the needle was still inside the lesion; (**D**) confirmation CT scan for checking the stamped area alignment with the actual lesion location; (**E**) thoracoscopic surgery using a uniportal approach; (**F**) the dye-stamped area was identified to guide thoracoscopic resection. CT, computed tomography; VATS, video-assisted thoracic surgery; HOR, hybrid operating room.

Under end-inspiratory breath-hold, an initial CBCT scan with a 4 s acquisition protocol (4s DynaCT Body) was obtained. The needle path was defined by marking the entry and target points of the needle, which was subsequently projected with a laser beam onto the skin of the patient (Figure 1A). Multi-joint arm-supporting systems (Unitrac^®^ Pneumatic Holding Arm, B. Braun) were used for precise control of the needle insertion, and a real-time fluoroscope with a progressive view was used to confirm needle advancement (Figure 1B). After the ablation needle was inserted, a confirmation scan was performed to check the final position of the ablation needle and the target lesion before initiating the ablation process (Figure 1C). Ablation was performed under contralateral lung ventilation, and the treated lung side was kept in apnea [41,42]. After completing the ablation based on the established protocol, CBCT was performed to check the ablation zone.

### 2.4. Image-Guided VATS

**Figure 2:**
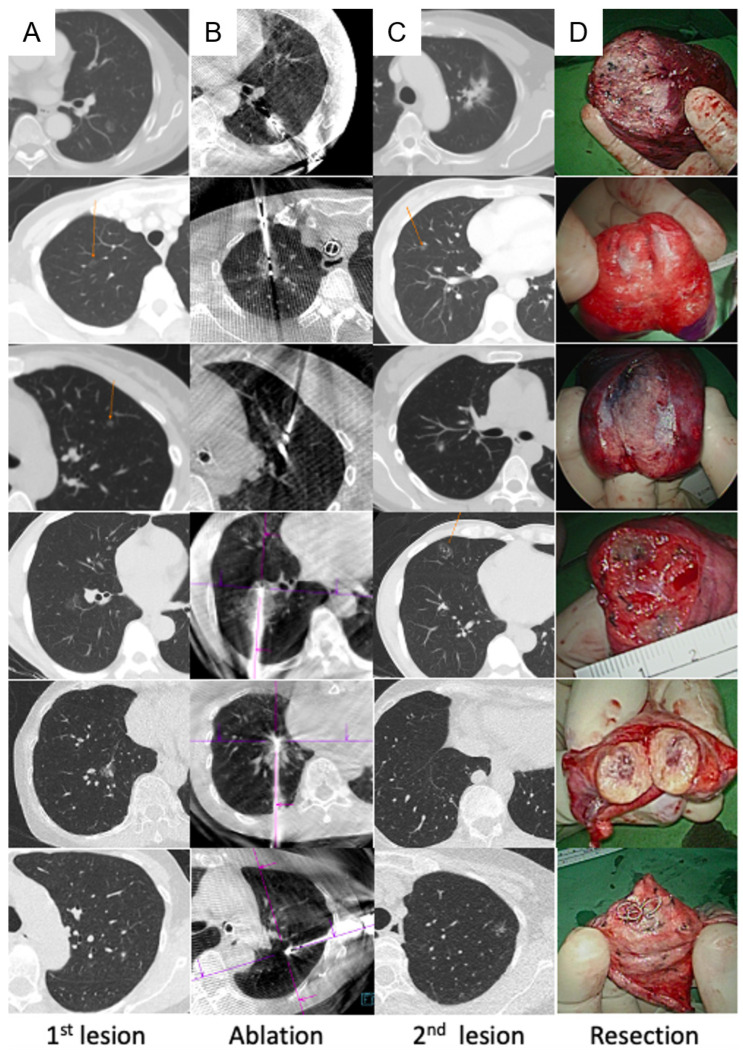
Demonstration of six cases from pre-ablation to resection. (**A**) Pre-ablation CT scan to indicate the location of the lesion to undergo ablation; (**B**) post-ablation CT showing the ablation zone and ablation needle in the zone; (**C**) CT to indicate the location of the lesion to undergo resection; (**D**) resected part of the lung showing that the lesion was successfully removed. CT, computed tomography.

Thoracoscopic surgery was performed after ablation, except in one patient, who underwent surgery first. Localization using the Artis Pheno system with different methods, including transbronchial [38] and transthoracic approaches [43,44], was performed before or during the thoracoscopic surgery, if necessary (Figure 1D). Uniportal video-assisted thoracic surgery (VATS) was routinely performed for simple wedge and anatomical lung resections (Figure 1E). Based on the thoracoscopic findings, the wedge resection was performed with the inclusion of a dye-containing area (Figure 1F) and/or centrally placed microcoils, which served as fiducial markers, as detected by intraoperative C-arm fluoroscopy. After wedge resection, the presence of the lesions was confirmed, and lymphadenectomy with nodal dissection or sampling was performed for suspected primary lung cancer. Additional pulmonary resection was performed if the section margin was inadequate (<tumor size), and a chest drainage tube was routinely placed. The patients were allowed to recover in the recovery room for observation. Figure 2 shows the images of the CT scans and resected lesions.

### 2.5. Postoperative Care

Following the completion of the procedure, all patients were kept in the recovery room for 1–2 h before returning to the general ward, and oral nonsteroidal analgesic agents and acetaminophen were administered once the patients resumed oral intake 2–4 h after the procedure. Roentgenograms of the chest were obtained at 6 h postoperatively and the following morning. All patients were examined in the outpatient department 7 days and 1 month after the procedure, and a chest roentgenogram was routinely performed on the same day.

### 2.6. Data Collection

Clinical data, operative findings, and pathological characteristics of the lung nodules were collected from medical records. The lesions were measured on the preoperative CT images. Lesion size was defined as the largest diameter observed in the axial view, and lesion depth was defined as the smallest distance from the center of the lesion to the pleura. The total accumulated radiation dose, expressed as the dose area product (DAP), was retrospectively calculated using data stored in the ARTIS workstation (Syngo X-Workplace; Siemens Healthcare GmbH, Erlangen, Germany). The durations of the procedures for ablation and surgical resection were recorded separately. The duration of ablation refers to the time from the initiation of the first CBCT scan to the conclusion of the last scan. The duration of surgery refers to the time from skin incision to the end of skin closure. The total anesthesia time was defined as the time between the start of anesthetic induction and extubation of the endotracheal tube.

### 2.7. Statistical Analysis

Descriptive statistics for continuous data are summarized as medians with interquartile ranges (IQRs) and means with standard deviations, whereas categorical data are presented as counts (percentages). All analyses were performed using SPSS version 20 software (IBM Corp., Armonk, NY, USA).

## 3. Results

We performed 49 procedures on 22 patients during the study period (Table 1). Patients were aged 36–68 years, and the majority (*n* = 18, 81.81%) were females. Each patient underwent at least one lesion ablation and lesion resection via VATS. The median lesion size and depth were 8.2 mm (IQR 7.1–11.3 mm) and 26.6 (IQR 21.3–37.4 mm), respectively, for the 24 lesions treated with MWA. The median duration of ablation was 48 min (IQR 32–68 min). Among the 24 ablated lesions, 2 were treated with cryoablation, and the remaining 22 were treated with microwave ablation. One patient underwent microwave ablation for two lesions located in the same lung.

The lesions treated with VATS had a median size of 8.9 mm (IQR 6.3–14.2 mm) and a depth of 10.5 mm (IQR 4.4–13.3 mm). The median duration of the VATS was 91.5 min (IQR 72–114 min). Among the 26 lesions, 5 were resected via segmentectomy, 1 via lobectomy, and the remaining 20 via wedge resection. Two patients underwent wedge resection and segmentectomy for two lesions located in the same lung. Another two patients underwent wedge resection for two lesions located in the same lung.

The operative findings and pathological characteristics are presented in Table 2. The median fluoroscopy duration was 2.5 min (IQR, 1.6–2.9 min), and the total DAP was 14,076 μGym^2^ (IQR, 11,764–22,354 μGym^2^). Among the 22 patients, 16 underwent nine or fewer DynaCT scans, whereas 6 underwent more than nine scans. The median global operation time was 227 min (IQR 196–249 min). The hospital length of stay was 1–3 days for 19 (86.36%) patients, whereas for 3 patients, it was 4, 8, and 6 days owing to complications, such as hemothorax and air leak. The needle biopsy findings for the lesions treated with ablation indicated adenocarcinoma (*n* = 2), benign alveolar parenchyma (*n* = 2), adenocarcinoma in situ (AIS) (*n* = 4), and atypical adenomatous hyperplasia (AAH) (*n* = 1). The pathological findings of the 26 resected lesions included adenocarcinoma (*n* = 7), AIS (*n* = 11), minimally invasive adenocarcinoma (MIA) (*n* = 4), AAH (*n* = 1), sclerosing pneumocytoma (*n* = 1), and metastatic colon cancer (*n* = 2). The median follow-up interval was 10.5 months (IQR 5–19 months). No patient experienced recurrence.

## 4. Discussion

In patients with multiple nodules, multiple-stage procedures are conventionally performed for various treatments. These management options include surgical excision in most cases, such as SBRT, RFA, or a combination of the above therapies [45]. It is important to consider complications, comorbidities, and compromised lung function before making treatment decisions. Performing the procedures at different times could involve patient exposure to general anesthetics at different times, as well as the potential risk of disease progression. Moreover, in the absence of an HOR for intraoperative imaging, patient transfer from the radiology room to the operating room can also involve the risk of complications and patient discomfort [46]. In some studies, SBRT has been used for multiple lesions synchronously or consecutively at 1-month intervals [47]. However, some cases of acute toxicity have been reported. A recent study demonstrated the preliminary outcomes of MWA and VATS in an HOR with some manageable complications [40]. These indicate the need for more advanced techniques to preserve the lung volume and minimize the risk of complications while providing patient comfort. Our early experience with VATS ablation has indicated that it is safe and minimally invasive, achieves tissue preservation, and can be individualized.

An HOR allows for one-step procedures while facilitating intraoperative image guidance and surgical intervention in a room. Single-stage augmented fluoroscopic bronchoscopy was performed under general anesthesia, followed by thoracoscopy.

Surgery is safe and feasible in an HOR [38]. Moreover, pleural stamping techniques for the localization of small pulmonary nodules before resection can be performed as a one-step procedure in an HOR [43,44]. In addition, percutaneous MWA is feasible in an HOR [41], where lung separation under general anesthesia can be efficiently performed with a lowered risk of complications [42]. Thus, there are many different management approaches for nodules with different features and specific requirements for which an HOR is favorable. For instance, if multiple nodules are located at different locations, one anterior and the other posterior, changing the patient’s position several times as needed in an HOR is convenient. Specific possibilities for a specific procedure are easily achievable in an HOR, for example, during procedures, such as a lobectomy, segmentectomy, or wedge resection. Considering these factors, our study combined ablation with VATS in an HOR equipped with CBCT guidance.

**Figure 3:**
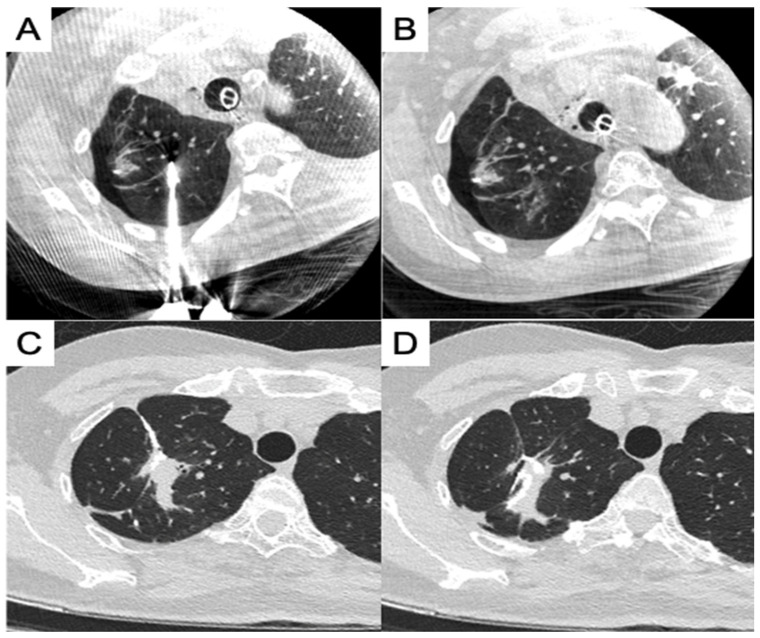
Combined ablation and wedge resection of the same pulmonary lobe. (**A**) Ablation placement after partial resection; (**B**) focal ablation zone away from the staple line; (**C**) post-procedure CT showed a staple line near the ablation zone; (**D**) post-procedure CT showed a staple line in the ablation zone.

Another concern during synchronous procedures is the sequence of approaches and deciding whether ablation or surgical resection should be performed first. In our study, we performed ablation for deeper lesions and surgical resection for peripheral pulmonary lesions. Ablation was performed first, because lung collapse was easily achievable before initiating VATS. Moreover, ablation demands better image quality for an efficient procedure, especially for small ground glass nodules, and in some cases, it requires a synchronous biopsy with a high accuracy for needle placement. Thus, the choice of the first procedure would depend on the physician’s experience and how the initial procedure would impact the latter, while simultaneously preparing for consequences and modifying as needed. Only one patient (no. 19) underwent surgical resection before the ablation of the same pulmonary lobe, and the deformed lobe owing to the wedge resection posed a challenge in recognizing the target area for placing the ablation needle (Figure 3A). However, tubular structures, such as pulmonary vessels and bronchi, around the target area could be traced to the division of the main trunks, and the area could be identified despite minor changes in the spatial relationship between the target and its surrounding structures (Figure 3B).

Bilateral sequential procedures for ablation and surgical resection are clinically challenging scenarios, and care should be taken for ventilation of the post-procedural lung during the procedure on the contralateral side, especially when no pleural drainage tube is placed on the post-procedural side. Once a pneumothorax exists, it can be aggravated during one-lung ventilation during the contralateral side procedure. In three cases involving bilateral procedures, we opted to perform ablation first because of the higher demand for image quality for lung ablation and because atelectasis during anesthesia could negatively impact successful ablation. Because we did not routinely perform chest drainage after ablation, we observed that for more than 5–10 min after one-lung ventilation, the lung shifted to the ablated side and no pneumothorax was detected under fluoroscopy, and the procedure was moved on to the surgical resection of the contralateral side. Although a chest drainage tube can be prophylactically placed on the ablation side, this is not mandatory if there is no evidence of a pneumothorax.

In this study, 11 patients underwent ablation and surgical resection of the same pulmonary lobe, and the resection and ablation zones were mostly completely separate. In some cases, the ablation zone for the central lesion can still be partially resected, along with another peripheral lesion that was surgically resected. The outcome was observed in the follow-up CT images (Figure 3C,D); however, no staple line leakage or prolonged air leak occurred in these cases.

This study has some limitations. First, there were very few bilateral lesions, which could have affected the results when comparing cases with multiple lesions located in one and both lungs. Most patients underwent MWA, with a few undergoing cryoablation, which may have been the reason for the varied procedure times in different patients. Future studies should consider bilateral lesions and a single form of ablation during synchronous procedures. All procedures were performed under GA in a single center, the sample size was small, and the study was retrospective. Future studies in multiple centers with larger numbers of participants are warranted.

## 5. Conclusions

Ablation with VATS under GA in an HOR is a minimally invasive procedure for patients with multiple pulmonary nodules. It is a safe technique with a minimal complication rate and a lower operation time, and it can be individualized. Future explorations with a larger number of patients and technical refinements are in progress and may lead to further success.

## Figures and Tables

**Table 1:** Patients and lesions characteristics.

No.	Sex	Age (Years)	1st Stage					Localization for VATS	2nd Stage				
			Location	Size	Depth	Procedure	Duration		Location	Size	Depth	Procedure	Duration
1	F	36	LUL	5.6	24.6	MWA	77	Transbronchial	RUL	1.1	0.5	Wedge	73
2	F	43	RUL	7.7	27.5	Cryo	60	Transbronchial	RLL	9.5	13.3	Segmentectomy	94
3	F	55	RUL	6.6	18.6	MWA	78	Transbronchial	RUL	5.8	9.4	Wedge	65
4	F	54	RUL	7.4	39.3	MWA	21	Transthoracic	RML	7.2	9.6	Wedge	58
5	F	68	RLL	15.6	61.3	MWA	57	-	RLL	14.2	6.0	Wedge	88
6	F	57	RUL	5.9	20.7	MWA	70	Transthoracic	RUL	7.3	4.2	Wedge	95
7	M	60	RLL	20.9	44.7	Cryo	49	-	RML	18.3	13.1	Wedge	89
8	M	42	RUL	6.7	30.5	MWA	71	Transthoracic	RUL × 2	12.4/6.3	14.5/6.7	Wedge × 2	127
9	F	68	LUL	6.2	21.9	MWA	47	Transthoracic	LUL	11.6	13.9	Wedge	75
10	F	58	RUL	7.4	39.3	MWA	24	-	RUL	14.8	15.5	Wedge	105
11	F	54	RUL	37.8	17.5	MWA	42	-	RML	8.2	4.0	Wedge	41
12	F	44	LLL	8.4	30.1	MWA	46	Transthoracic	LLL	6.0	3.6	Wedge	71
13	F	43	RUL/RLL × 2	9.4 13.3/11.1	35.5 30.8/18.9	MWA	78	Transthoracic	RLL	9.4	12.2	Wedge	120
14	F	63	RLL	7.8	23.9	MWA	61	Transthoracic	RUL	5.3	3.1	Wedge	30
15	F	33	LUL	5.1	22.2	MWA	30	Transthoracic	LLL × 2	8.4/5.8	4.4/11.1	Wedge × 2	118
16	M	57	RML	10.3	25.2	MWA	18	-	RUL	36.6	9.8	Lobectomy	118
17	F	64	LUL	8.9	31.4	MWA	45	-	LUL/LLL	14.4/14.1	13.1/35.5	Wedge/Segmentectomy	172
18	F	43	RUL	8.1	52.1	MWA	68	Transthoracic	RUL	6.6	4.1	Wedge	38
19	F	65	RUL	8.1	3.4	Wedge	50	Transthoracic	RUL	4.8	27.6	MWA	88
20	M	52	RUL	10.4	40.4	MWA	38	Transthoracic	RLL/RLL	6.3/10.2	12.2/7.2	Wedge/Segmentectomy	170
21	F	66	RUL	13.3	19.2	MWA	31	Transbronchial	LUL	30.2	15.7	Segmentectomy	102
22	F	68	RUL	11.4	25.7	MWA	32	Transbronchial	LLL	40.8	12.8	Segmentectomy	110
Median		56		8.2	26.6		48			8.9	10.5		91.5
IQR		21		7.1–11.3	21.3–37.4		32–68			6.3–14.2	4.4–13.3		72–114

Cryo, cryoablation; F female; IQR, interquartile range; LLL, left lower lobe; LUL, left upper lobe; M, male; MWA, microwave ablation; RLL, right lower lobe; RML, right middle lobe; RUL, right upper lobe.

**Table 2:** Details of operative procedures and postoperative results.

No.	Fluoroscopy Duration (min)	Number of Dyna CT Scans	Total DAP (μGym^2^)	Total Ana Time	Global OR Time	Complications	LOS	Histology		Postoperative Follow-Up Interval (mo)	Evidence of Residual Disease or Recurrence
								Needle Biopsy	Resection		
1	2.7	14	12,449	210	220	-	1	-	Adenocarcinoma	27	No
2	2.8	7	17,436	222	232	-	2	-	MIA	26	No
3	4.7	8	32,943	223	229	-	1	-	AIS	22	No
4	1.9	6	6243	157	162	-	1	-	AIS	22	No
5	2.7	9	14,032	227	240	-	3	AIS at least	Sclerosing pneumocytoma	20	No
6	2.7	9	16,095	233	243	-	2	Benign alveolar parenchyma	AIS	19	No
7	1.2	6	5416.3	202	218		1	-	Adenocarcinoma	18	No
8	5.1	9	32,305	205	214	Hemothorax	8	-	AIS × 2	17	No
9	2.7	12	13,794	225	235	-	2	-	Adenocarcinoma	15	No
10	2.2	6	14,119	193	196	-	2	-	Adenocarcinoma	15	No
11	1.6	5	11,764	162	180	-	2	-	Adenocarcinoma	10	
12	1.6	9	8871.2	169	182	-	2	AIS at least	AIS	11	No
13	2.2	14	36,430	264	277	Prolonged air leak	6	-	Metastatic colon cancer	8	
14	3.2	12	7514.5	172	192	-	2	Benign alveolar parenchyma	AIS	10	No
15	2.9	10	17,422	196	210	-	2	Adenocarcinoma	AIS/AAH	9	No
16	1.7	9	30,818	242	253	-	2	AIS at least	Adenocarcinoma	6	No
17	1.2	8	22,704	296	302	-	2	-	MIA/MIA	5	No
18	1.2	9	11,878	162	185	-	1	AAH	AIS	4	No
19	1.6	10	17,482	216	226	-	3	-	AIS	3	No
20	1.8	8	13,042	281	291	-	2	-	AIS/MIA	3	No
21	3.3	8	5566.8	375 *	380 *	-	4	-	Metastatic colon cancer	1	No
22	5.2	9	22,354	240	249	-	3	AIS at least	Adenocarcinoma	1	No
Median	2.5	9	14,076	219	227		2			10.5	
IQR	1.6–2.9	8–10	11,764–22,354	193–240	196–249		2–3			5–19	

* AAH, atypical adenomatous hyperplasia; AIS, adenocarcinoma in situ; DAP, dose area product; LOS, length of postoperative stay; MIA, minimally invasive adenocarcinoma. * Patient no. 21 underwent an additional abdominal surgery during the same period.

## Data Availability

The data presented in this study are available in this article.
